# Increased death and exhaustion of CD69^high^ T cells and NK cells are associated with PD-1 antibody application in the *in vitro* co-culture system

**DOI:** 10.7717/peerj.15374

**Published:** 2023-05-08

**Authors:** Ying Wang, Zhengyi Sun, Xue Du, Qiuyang Yu, Chao Sun, Jing Huang, Liying Wang

**Affiliations:** 1 Department of Laboratory Medicine, The First Hospital of Jilin University, Changchun, China; 2 Cancer Centre, The First Hospital of Jilin University, Changchun, China; 3 Institute of Pediatrics, The First Hospital of Jilin University, Changchun, China

**Keywords:** PD-1 monoclonal antibody, Acquired resistance, T cells, Natural killer cells, Tumor microenvironment

## Abstract

**Background:**

The application of PD-1 monoclonal antibody (mAb) helps to treat non-small cell lung cancer, but acquired resistance has emerged in clinical practice. We tested the hypothesis that acquired resistance of anti-PD-1 immunotherapy is linked to death and exhaustion of activated T and NK cell.

**Methods:**

The co-culture system of HCC827 cells and peripheral mononuclear cells (PBMCs) was established to evaluate the effect of PD-1 mAb on the death rate and exhaustion of T and NK cell. The predisposing role of CD69 for death and exhaustion was validated by using PHA-activated PBMCs of CD69^low^ NSCLC patients. The 10-colour/three laser flow cytometer was used to test related markers for cell activation, death and exhaustion.

**Results:**

We found that PD-1 mAb increase the death and exhaustion of T cells and NK cells in a dose-dependent way when PBMCs from NSCLC patients whose the percentages of CD69^+^ cells in peripheral blood T cells were greater than 5% (CD69^high^ NSCLC patients). By analyzing PBMCs from healthy volunteers and CD69^low^ NSCLC patients, we found that T cells and NK cells can be induced to die by PD-1 mAb after PHA activation, and had a tendency to raise the rate of cell exhaustion.

**Conclusions:**

Our findings imply that increased death and exhaustion of CD69^high^ T cells and NK cells are associated with ineffective anti-PD-1 immunotherapy in lung cancer. The CD69 expression of T cells and NK cells may be developed as a potential predictor for acquired resistance of anti-PD-1 immunotherapy. These data may provide ideas to guide individualized medication of PD-1 mAb in NSCLC patients.

## Introduction

Non-small cell lung cancer (NSCLC) is one of the malignant diseases with high morbidity (Kaur et al., 2021). Since most patients with advanced NSCLC lose the opportunity for surgical resection, they often require anti-tumor immunotherapy, such as anti-programmed cell death-1 (PD-1) immunotherapy (Shepherd, Douillard & Blumenschein, 2011). However, some of them do not benefit from the targeted PD-1 immunotherapy in clinic. Some responders relapse after a period of time, suggesting the emergence of acquired resistance (Pirozyan et al., 2020; Wang & Wu, 2020). It is important to elucidate related mechanism to increase the efficacy of anti-PD-1 immunotherapy.

It has been reported that the low density of tumor infiltrating lymphocytes (TILs), especially T cells and NK cells, may be associated with acquired resistance of anti-PD-1 immunotherapy (Sevenich, 2019; Kawashima et al., 2021). A possible explation for the association is insufficient recruitment of immune cells (Bonaventura et al., 2019). However, some researchers have also found that the TILs in tumor microenvironment (TME) not only undergo continuous proliferation, but also death (Zhu, Petit & Van den Eynde, 2019). The death of the TILs restrains the amount of lymphocytes in TME, resulting in poor disease prognosis (Cristescu et al., 2018; Horton et al., 2018). So far, the correlation between the death rate of the TILs and the acquired resistance to the anti-tumor immunotherapy with PD-1 monoclonal antibody (mAb) is still unclear. Besides, exhaustion of lymphocytes is considered as another pathway for acquired resistance of anti-PD-1 immunotherapy (Wherry & Kurachi, 2015). T cell immunoglobulin and ITIM domain (TIGIT), a lymphocyte exhaustion-related molecule, is mainly expressed on T cells and NK cells and has been confirmed to be associated with the acquired resistance to PD-1 blockade (Salas-Benito, Eguren-Santamaria & Sanmamed, 2021; Harjunpää & Guillerey, 2020).

CD69 is known to be an activation marker of lymphocytes and a crucial regulator of various immune responses (Sancho, Gómez & Sánchez-Madrid, 2005). As a membrane receptor, CD69 plays an important role in migration of activated lymphocytes by interacting with its ligands S1PR1 (Sphingosine 1-phosphate receptor 1) or Myl9/12 (Myosin light chains 9, 12a and 12b) (Bankovich, Shiow & Cyster, 2010; Hayashizaki et al., 2016). Moreover, CD69 is related to ketamine-induced apoptosis *in vitro* and can also control the exhaustion of TILs in murine breast cancer model (Mita et al., 2018; Zhou et al., 2018). The recent research about neuroendocrine neoplasms found a negative correlation between progression-free survival after anti-PD-1 immunotherapy and expression of the CD69 activation marker on naïve T and NK cells in pre-treatment peripheral blood samples (MacFarlane et al., 2021). Another study reported that the proportion of peripheral NK cells expressing CD69 significantly increased in NSCLC patients with higher tumor stages compared with stage I and the healthy controls (Seier et al., 2022). However, no study investigated the relationship between expression level of CD69 on lymphocytes and the acquired resistance of anti-PD-1 immunotherapy in NSCLC.

In this study, the co-culture system of HCC827 cells and peripheral mononuclear cells (PBMCs) *in vitro* was used to evaluate the concentration effect of PD-1 mAb on the death rate and exhaustion of lymphocytes. We focused attention on whether PD-1 mAb promoted the death and exhaustion of lymphocytes in TME of NSCLC and tried to identify the influence of activated lymphocytes (CD69^high^ expression) in the process. And phytohemagglutinin (PHA) was then used to activate PBMCs from CD69^low^ NSCLC patients and healthy volunteers to verify the predisposing role of CD69^high^ expression for death and exhaustion of lymphocytes in PD-1 mAb immunotherapy.

## Materials and Methods

### Samples

Peripheral blood samples were obtained from healthy individuals and NSCLC patients. All participants were recruited at the First Hospital of Jilin University, Changchun, China, from October 2021 to September 2022. The study was approved by the Human Ethics Committee of the First Hospital of Jilin University (22K056-002). The written informed consents were obtained from all participants included in the study.

### PD-1 mAb

Sintilimab (Cat#IB1308; Innovent Biologics, Suzhou, China), a highly selective and fully humanised mAb, blocks the interaction between PD-1 and its ligands (Shi et al., 2019). It is approved in China for the treatment of NSCLC (Zhang et al., 2022).

### Establishment of *in vitro* model system of anti-tumor therapy

To mimic the effect of PD-1 mAb on lymphocytes in TME of NSCLC, we established an *in vitro* model system of anti-tumor therapy. All experiments were performed with mycoplasma-free cells. In the model system, there were three parts, including human lung cancer cell line HCC827 cells (RRID: CVCL_2063, Shanghai Zhong Qiao Xin Zhou Biotechnology Co., Shanghai, China, Cat#ZQ0386), PD-1 mAb and PBMCs or PHA activated PBMCs. Firstly, HCC827 cells were seeded into 24-well plates with RPMI1640 medium containing 10% fetal bovine serum (FBS; Thermo fisher scientific, Waltham, MA, USA, Cat#10099) and cultured for 3 h to adhere wall. Secondly, PBMCs were isolated from peripheral blood of healthy volunteers or NSCLC patients through density gradient centrifugation (Ficoll-Paque; Sigma-Aldrich, St. Louis, MI, USA, Cat#10771). According to the requirement of activation, PBMCs were treated with or without 2 ug/ml PHA (Cat#40110ES08; Yesen Biotechnology, Shanghai, China) for 24 h. After centrifugation for 5 min at 500×g, PBMCs were washed with PBS and then inoculated into HCC827 cell culture wells in a ratio of 2:1 (3 × 10^5^ cells/well). Thirdly, PD-1 mAb were added into the co-culture system. To clarify the dose effect of PD-1 mAb, the gradually increasing concentration of PD-1 mAb were added for performing a concentration curve analysis. According to the concentration curve, 1 mg/ml of PD-1 mAb presented the maximal effect on death (Annexin V) and exhaustion (TIGIT) of T cells and NK cells in TME, and was selected for subsequently observing the effect of PD-1 mAb after PBMC activation. After 24 h of adding PD-1 mAb, the cells and the culture supernatants were harvested and analyzed by flow cytometry.

#### Flow cytometry analysis

Samples were tested by 10-colour/three laser flow cytometer (FACSCanto^™^; BD Bioscience, Franklin Lakes, NJ, USA) and analyzed by BD FACSDiva^™^ software (BD Bioscience, Franklin Lakes, NJ, USA). The following fluorescently-conjugated antibodies were used for cell phenotypic analysis: CD45- PerCP (Cat#652803; BD Bioscience, Franklin Lakes, NJ, USA), CD45-V500-c (Cat#662912; BD Bioscience, Franklin Lakes, NJ, USA), CD3-FITC (Cat#349201; BD Bioscience, Franklin Lakes, NJ, USA), CD3-APC-H7(Cat#663490; BD Bioscience, Franklin Lakes, NJ, USA), CD19-APC(Cat#652804; BD Bioscience, Franklin Lakes, NJ, USA), CD20-PE(Cat#555623; BD Bioscience, Franklin Lakes, NJ, USA), TIGIT-BV421(Cat#747844; BD Bioscience, Franklin Lakes, NJ, USA), CD69-FITC(Cat#555530; BD Bioscience, Franklin Lakes, NJ, USA). All antibodies for flow cytometry in this study were purchased from BD Biosciences. The death of T/NK cells was measured by staining with Annexin V-PE antibody (Cat#559763; BD Bioscience, Franklin Lakes, NJ, USA). The gating strategy for cell subsets were shown in corresponding figures. Due to the occupancy of fluorescent channels, CD19-APC antibody or CD20-PE antibody was used to mark B cells (Blüml et al., 2013; Cascino, Roederer & Liechti, 2020). The similarity of CD19^+^ B cells and CD20^+^ B cells in peripheral blood leukocytes from NSCLC patients had been confirmed by using flow cytometry analysis (Fig. S1). In addition, according to the strategies in this study, the CD45^+^CD3^−^CD19^−^ cells or CD45^+^CD3^−^CD20^−^ cells could be counted as NK cells. Almost all CD45^+^CD3^−^CD19^−^/CD20^−^ cells were equivalent to CD45^+^CD3^−^CD56^+^/CD16^+^ NK cells by using CD45^high^SSC^low^ phenotype to gate lymphocytes (Fig. S1).

### Statistical analysis

Differences among normally distributed variables were analyzed using a Student’s *t*-test. For variables that were not normally distributed, a Mann-Whitney U test or Kruskal-Wallis ANOVA test was used. Statistical significance is shown as: *P < 0.05, **P < 0.01, ***P < 0.001. Statistical analyses were performed using Prism 5.0 (RRID:SCR_002798; GraphPad Prism, San Diego, CA, USA) and SPSS 21.0 statistical software package (RRID:SCR_002865; SPSS, Chicago, IL, USA).

## Results

### PD-1 mAb promoted the death and exhaustion of T cells and NK cells in the *in vitro* co-culture system of HCC827 cells and PBMCs from NSCLC patients

**Figure 1:**
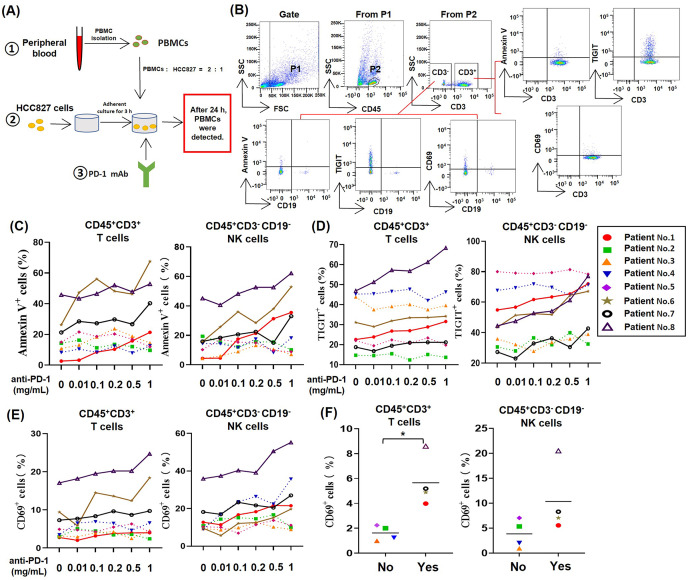
For partial NSCLC patients, the death and exhaustion of T cells and NK cells in TME increased with increasing concentration of PD-1 mAb. (A) Schematic diagrams of the co-culture system of HCC827 cells and PBMCs *in vitro*. (B) Flow-cytometry dot plots show the strategy for gating T cell subsets and NK cells with Annexin V^+^, TIGIT^+^ and CD69^+^ phenotype. (C–E) PBMCs were isolated from peripheral blood of NSCLC patients and inoculated into HCC827 cell culture wells with gradually increasing concentration of PD-1 mAb for 24 h. The expression of (C) Annexin V, (D) TIGIT and (E) CD69 were analyzed on CD45^+^CD3^+^ T cells and CD45^+^CD3^−^CD19^−^ NK cells. The same shape represents the same patient. (F) The expression of CD69 in peripheral blood of NSCLC patients were analyzed on CD45^+^CD3^+^ T cells and CD45^+^CD3^−^CD19^−^ NK cells. Yes, T cells and NK cells of NSCLC patients were prone to death and exhaustion after the treatment with PD-1 mAb. No, T cells and NK cells of NSCLC patients weren’t prone to death and exhaustion after the treatment with PD-1 mAb. Student’s paired t-test, *P < 0.05.

To study the effect of PD-1 mAb on lymphocytes in TME of NSCLC, we established an *in vitro* model system of anti-tumor therapy. In this system, we co-cultured the human NSCLC cell line HCC827 cells and PBMCs from NSCLC patients with different concentrations of PD-1 mAb (Fig. 1A), and analyzed the death rates and exhaustion of T cells and NK cells in the co-culture system by gating CD45^+^CD3^+^ cells and CD45^+^CD3^−^CD19^−^ cells, respectively (Fig. 1B). The result showed that PD-1 mAb increased the death rates of T cells (CD45^+^CD3^+^ Annexin V^+^) and NK cells (CD45^+^CD3^−^CD19^−^ Annexin V^+^) of several NSCLC patients (patient No. 1, and No. 6-8) in a dose-dependent way (Fig. 1C and Fig. S2). Similarly, PD-1 mAb could also significantly up-regulate the levels of TIGIT on T cells (CD45^+^CD3^+^TIGIT^+^) and NK cells (CD45^+^CD3^−^CD19^−^ TIGIT^+^) of these four patients (Fig. 1D and Fig. S2). However, for other four patients (patient No. 2-5), PD-1 mAb had no promoting effect on the death rates and exhaustion of T cells and NK cells in the co-culture system (Figs. 1C and 1D). By analyzing CD69^+^ subets in CD45^+^CD3^+^ and CD45^+^CD3^−^CD19^−^ cells, we found PD-1 mAb could increase the percentages of CD69^+^ T cells and CD69^+^ NK cells in the co-culture system containing PBMCs of patient No.1 and No.6-8 in a dose-dependent way (Fig. 1E). Besides, by observing the percentages of CD69^+^ cells in the peripheral blood lymphocytes of all eight NSCLC patients, we found that the percentages of CD69^+^ T cells and CD69^+^ NK cells were significantly higher in patients whose T/NK cells affected by PD-1 mAb (Yes) than those not affected by PD-1 mAb (No) (Fig. 1F). These results suggest that the activation status (CD69 positive) of T cells and NK cells in the peripheral blood of NSCLC patients may be closely related to PD-1 mAb induced death and exhaustion of T cell and NK cell in TME.

### PD-1 mAb induced the death and exhaustion of T cells and NK cells from CD69^high^ NSCLC patients

**Figure 2:**
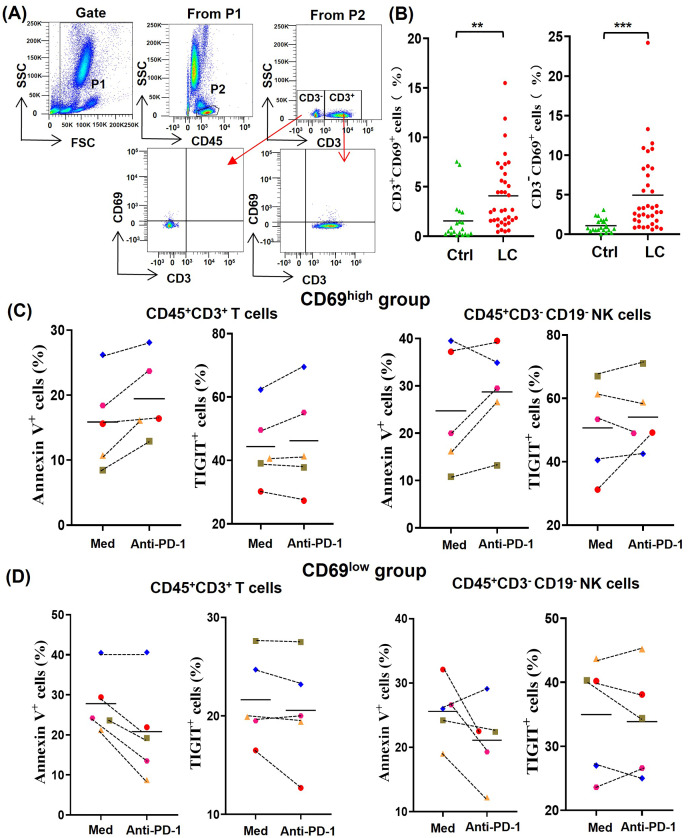
For CD69^high^ NSCLC patients, PD-1 mAb induced the death and exhaustion of the T cells and NK cells when co-cultured with tumor cells. (A) Flow-cytometry dot plots show the strategy for gating peripheral blood lymphocyte cells with CD69^+^ phenotype. (B) The expression of CD69 in peripheral blood of healthy volunteers (Ctrl, *n* = 20) and NSCLC patients (LC, *n* = 36) were analyzed on CD45^+^CD3^+^ T cells and CD45^+^CD3^−^ lymphocyte cells. Student’s paired t-test, **P < 0.01 ***P < 0.001. (C and D) PBMCs were isolated from peripheral blood of NSCLC patients and inoculated into HCC827 cell culture wells with (anti-PD-1) or without (Med) 1 mg/ml PD-1 mAb for 24 h. The expression of Annexin V and TIGIT were analyzed on CD45^+^CD3^+^ T cells and CD45^+^CD3^−^CD19^−^ NK cells in the (C) CD69^high^ group and (D) CD69^low^ group. The CD69^high^ group means NSCLC patients with the percentages of CD69^+^ cells in T lymphocytes were greater than 5%. The CD69^low^ group means NSCLC patients with the percentages of CD69^+^ cells in T lymphocytes were less than 5%. The same shape represents the same patient.

In order to understand the difference of activated lymphocytes in peripheral blood between lung cancer patients and healthy person, we analyzed the percentages of CD69^+^ lymphocytes by gating CD45^+^CD3^+^CD69^+^ T cells and CD45^+^CD3^-^CD69^+^ non-T cells, respectively (Fig. 2A). By comparing the percentages of CD45^+^CD3^+^CD69^+^ cells (CD69^+^ T cells) and CD45^+^CD3^−^CD69^+^ cells (CD69^+^ non-T cells) in peripheral blood of 20 healthy volunteers with that of 36 NSCLC patients, we found that the percentages of CD69^+^ T cells and CD69^+^ non-T cells in peripheral blood of NSCLC patients were significantly higher than that of healthy volunteers (Fig. 2B). In order to determine the effect of the activation state in peripheral blood lymphocytes on anti-PD-1 tumor immunotherapy, we randomly selected five NSCLC patients whose the percentages of CD69^+^ cells in peripheral blood T cells were greater than 5% (CD69^high^ group) and five NSCLC patients whose the percentages of CD69^+^ cells in peripheral blood T cells were less than 5% (CD69^low^ group). By analyzing the percentages of Annexin V^+^ T/NK cells and TIGIT^+^ T/NK cells, we found that PD-1 mAb were prone to promote the death and exhaustion of T cells and NK cells in the CD69^high^ group (Fig. 2C). However, PD-1 mAb were prone to reduce the death and exhaustion of T cells and NK cells in the CD69^low^ group (Fig. 2D). These results suggest that some lung cancer patients have CD69^high^ T cells and NK cells in their peripheral blood, and these CD69^high^ cells are prone to death and exhaustion after PD-1 mAb therapy.

### Analysis of PHA-activated PBMC model by using peripheral blood of healthy volunteers and NSCLC patients

**Figure 3:**
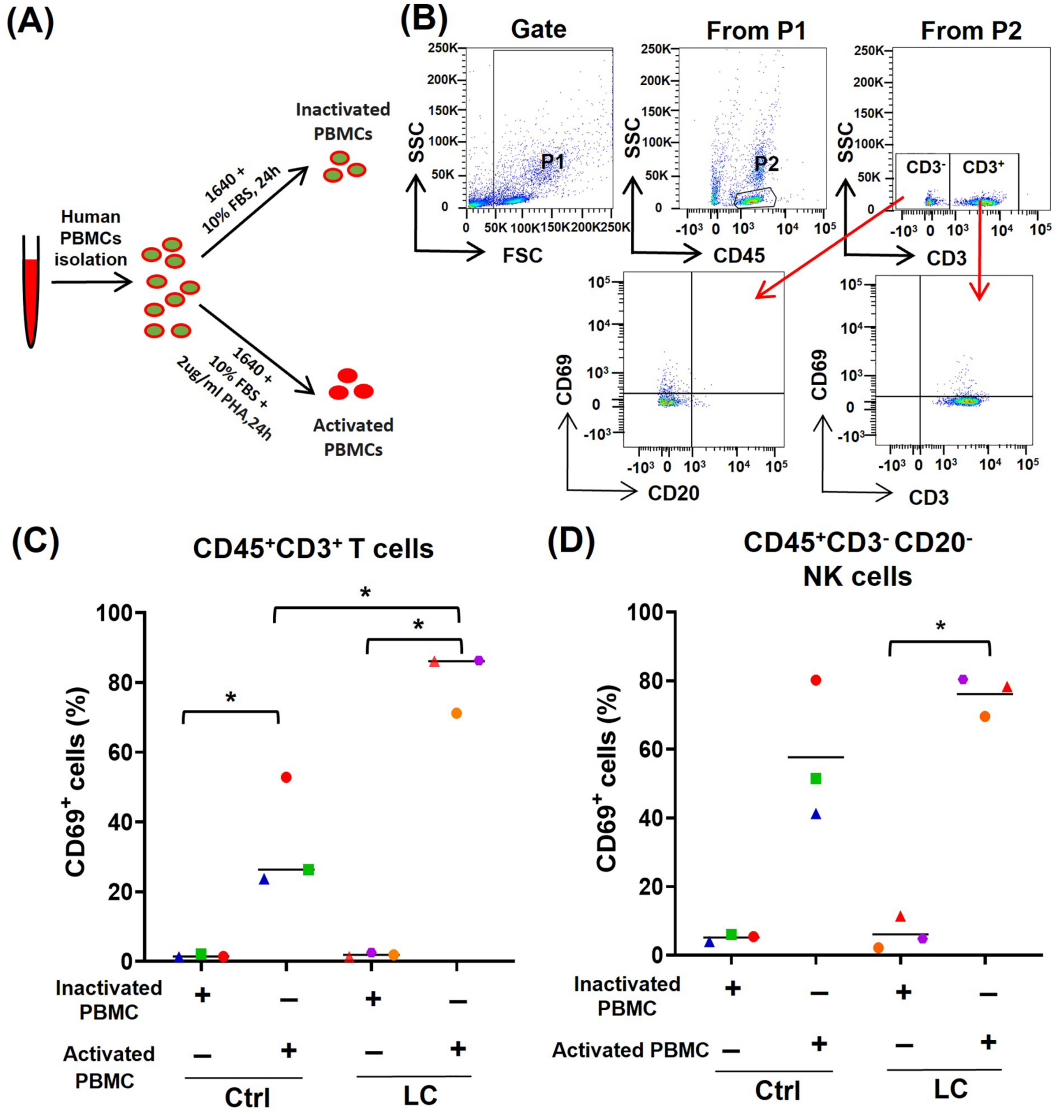
Analysis of PBMC activation model by using peripheral blood of healthy volunteers (Ctrl) and NSCLC patients (LC). (A) Schematic diagrams of PBMC activation model *in vitro*. (B) Flow-cytometry dot plots show the strategy for gating PBMCs with CD69^+^ phenotype. (C and D) The expression of CD69 in inactivated or PHA activated PBMCs of the Ctrl group and LC group were analyzed on (C) CD45^+^CD3^+^ T cells and (D) CD45^+^CD3^−^CD20^−^ NK cells. The same shape represents the same patient. Student’s paired t-test, *P < 0.05.

To further determine whether the activated (CD69^high^) T cells and NK cells were prone to death and exhaustion after using PD-1 mAb, we used PHA to activate PBMCs from either healthy volunteers (*n* = 3) or NSCLC patients (*n* = 3) (Fig. 3A) and analyzed the activation status of T cells and NK cells by gating CD45^+^CD3^+^CD69^+^ cells and CD45^+^CD3^−^CD20^−^CD69^+^ cells, respectively (Fig. 3B). On CD45^+^CD3^+^T cells in PHA-activated PBMCs, the expression of CD69 was significantly up-regulated (Fig. 3C). The percentages of CD45^+^CD3^+^CD69^+^ cells were increased from 1.61% to 34.3% in healthy volunteers’ group and from 1.9% to 81.2% in the NSCLC patients’ group. The percentages of CD45^+^CD3^+^CD69^+^ cells in the NSCLC patients’ group were significantly higher than that in healthy volunteers’ group (Fig. 3C). On CD45^+^CD3^−^CD20^−^ NK cells, PHA could increase the percentage of CD69^+^ cells from 6.1% to 76.1% in the NSCLC patients’ group, and trend to raise the percentage of CD69^+^ cells in the healthy volunteers’ group (Fig. 3D). These results suggest that the PBMCs from NSCLC patients are more easily activated by PHA, compared with that from healthy volunteers.

### PD-1 mAb induced the death of PHA-activated T cells and NK cells from PBMCs of CD69^low^ NSCLC patients

**Figure 4:**
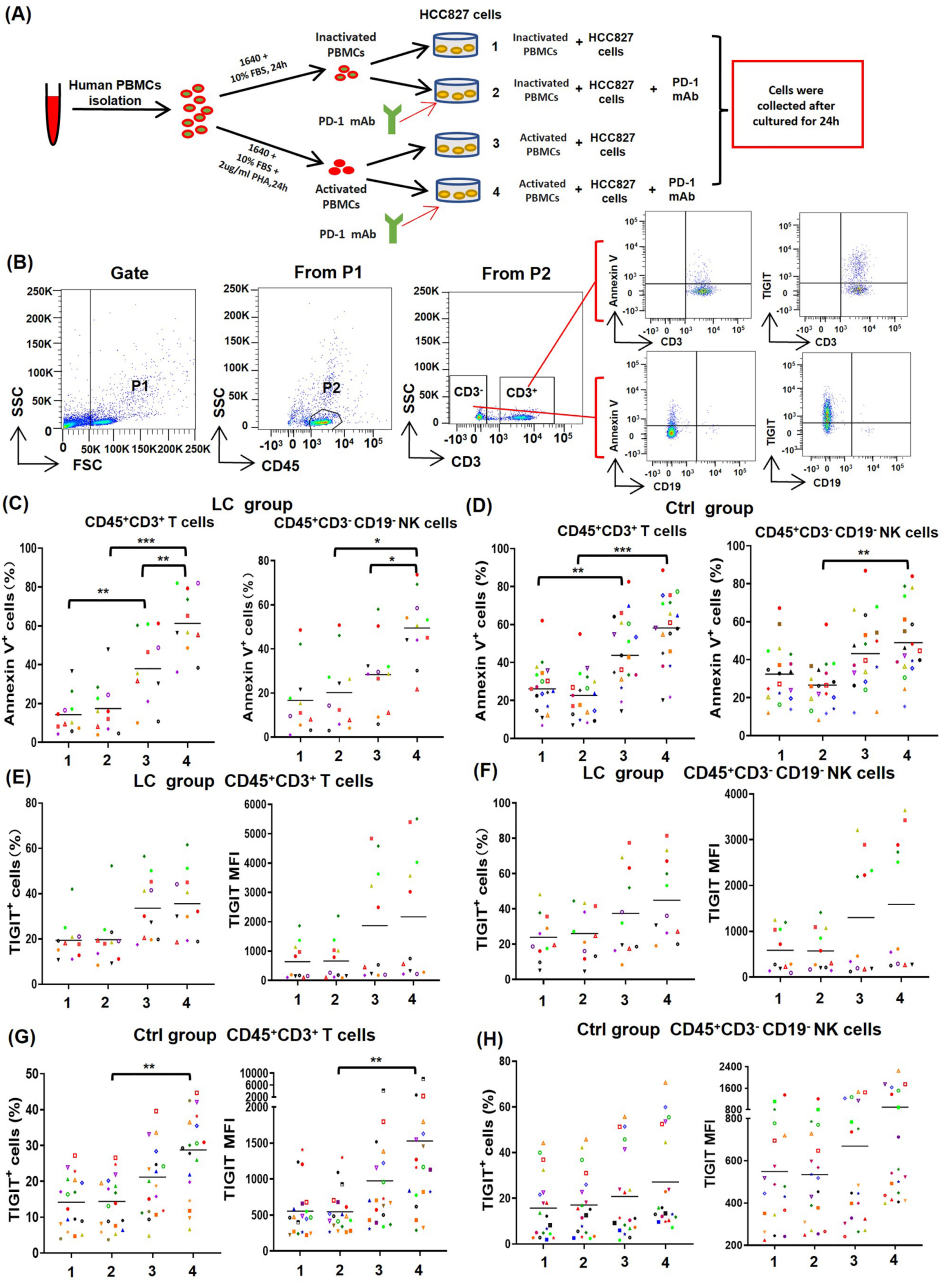
Effects of PD-1 mAb on the death and exhaustion of the T cells and NK cells when PBMCs co-cultured with tumor cells. (A) Schematic diagrams of different types of co-culture system *in vitro*. (B) Flow-cytometry dot plots show the strategy for gating T cells and NK cells with Annexin V^+^ and TIGIT^+^ phenotype. (C-D) The death rate of CD45^+^CD3^+^ T cells and CD45^+^CD3^−^CD19^−^ NK cells in the (C) LC group (*n* = 11) and (D) Ctrl group (*n* = 19). (E and F) Proportions and MFI of TIGIT on (E) CD45^+^CD3^+^T cells and (F) CD45^+^CD3^−^CD19^−^ NK cells in the LC group (*n* = 11). (G and H) Proportions and MFI of TIGIT on (G) CD45^+^CD3^+^T cells and (H) CD45^+^CD3^−^CD19^−^ NK cells in the Ctrl group (*n* = 19). The same shape represents the same patient. Student’s paired t-test, *P < 0.05, **P < 0.01 ***P < 0.001.

To determine the effect of PD-1 mAb on activated T cells and NK cells, PBMCs from NSCLC patients (*n* = 11) and healthy volunteers (*n* = 19) were cultured in four different types of *in vitro* co-culture system (Fig. 4A), respectively. The gating strategy was presented in Fig. 4B. By analyzing results of CD69^low^ NSCLC patients (LC group, CD69^low^ means the percentages of CD69^+^ cells in peripheral blood T cells are less than 5%), we found that the death rates of T cells (CD45^+^CD3^+^Annexin V^+^) and NK cells (CD45^+^CD3^−^CD19^−^Annexin V^+^) in the co-culture system containing PHA-activated PBMCs with or without PD-1 mAb were all significantly increased (Fig. 4C). When PD-1 mAb was added in the co-culture system, the death rates of PHA-activated T cells and NK cells further reached to 61.3% and 49.5%, respectively. But the addition of PD-1 mAb had no effect on the death rates of non-activated T cells and NK cells (Fig. 4C). By analyzing healthy volunteers (Ctrl group), we found that the death rates of PHA-activated T cells and NK cells were also significantly up-regulated, but PD-1 mAb only trended to raise the death rates of T/NK cells in the PHA-activated group (Fig. 4C and Fig. S3). We also analyzed the percentages of TIGIT^+^ T/NK cells and expression levels (mean fluorescence intensity, MFI) of TIGIT on T/NK cells in the LC group and Ctrl group. The result showed that PD-1 mAb only trended to raise the percentage of TIGIT^+^ T/NK cells and the MFI of TIGIT on T/NK cells in both the LC group (Figs. 4E and 4F) and Ctrl group (Figs. 4G, 4H and Fig. S3), although PHA could increase the percentage of TIGIT^+^ T cells and the MFI of TIGIT on T cells in the Ctrl group (Fig. 4G). These results suggest that the activated PBMCs are more likely to die and exhaust in TME, which can be promoted by PD-1 mAb.

## Discussion

In this study, we found that PD-1 mAb promoted the death and exhaustion of T cells and NK cells with a dose-dependent way in the *in vitro* co-culture system of HCC827 cells and PBMCs from NSCLC patients, as shown in Figs. 1C and 1D. It seems that the accumulated PD-1 mAb might be responsible for the recurrence of NSCLC in some responders by inducing the death and exhaustion of T cells and NK cells in TME. These results are consistent with the recommendations of clinical guidelines for treating NSCLC patients. The guidelines point out that clinicians should pay attention to the long half-life of immune checkpoint inhibitors (National Comprehensive Cancer Network, 2021). According to the instruction of the application of PD-1 mAbs in clinic, the approved PD-1 mAbs have a half-life ranging from 5 days to 20 days or more, it is recommended for lung cancer patients with intravenous drip of PD-1 mAb (1, 3 or 10 mg/kg) every two weeks or three weeks (Lee et al., 2018; Mo et al., 2018; Patnaik et al., 2015; Wrangle et al., 2018). Based on these evidences, PD-1 mAbs may gradually accumulate *in vivo* in the process of the anti-PD-1 immunotherapy with a fixed time cycle. From this perspective, NSCLC patients who possibly represent acquired resistance to PD-1 mAb immunotherapy should be screened in early stage and the clincians are necessary to reconsider the application strategy of PD-1 mAbs for these patients.

As an activation marker, CD69 is generally considered as the biomarker of enhanced anti-tumor activities of effector lymphocytes (Alotaibi et al., 2020). However, we found that there is a positive correlation between the percentages of CD69^+^ T/NK cells of NSCLC patients and the death and exhaustion of T /NK cells caused by PD-1 mAb *in vitro*, as shown in Figs. 2C and 2D. Similarly, T cells and NK cells in PBMCs of CD69^low^ NSCLC patients could be induce to die by PD-1 mAb after PHA activation, and had a tendency to raise the rate of cell exhaustion, as shown in Figs. 4C, 4E and 4F. Consistent with our results, some researchers found that anti-CD69 antibody can potentiate antitumor efficacy of dendritic cell-based vaccine, which is considered to be associated with increased T-cell proliferation and activity (Wei et al., 2017). Others also found that CD69 deficient mice challenged with prostate carcinoma showed greatly reduced tumor growth and prolonged survival, which be due to the enhanced anti-tumor response of lymphocytes and increased lymphocytes in local (Esplugues et al., 2003). All these studies imply that CD69 play a negatively regulated role in anti-tumor immune responses.

In Figs. 2C and 4C, our results show that activated T/NK cells could be induced to die by PD-1 mAb in the *in vitro* co-culture system. However, PD-1 mAb cannot induce the death of activated lymphocytes in the absence of tumor cells, as shown in Fig. S4. The PD-1 induced death of TILs seem to be related to the presence of tumor cells. Recent studies revealed intrinsic expression of PD-1 in lung cancer cells (Yao et al., 2018). In an NSCLC patient expressing tumor-intrinsic PD-1, the tumor rapidly progressed upon anti-PD-1 therapy for 2 months (Du et al., 2018). Although the exact mechanisms remain unclear, we speculate that blockade of NSCLC-intrinsic PD-1 can release PD-L1 and stimulate the expression of co-inhibitory molecules such as Fas ligand, which jointly mediate the apoptotic pathway of activated lymphocytes after application of PD-1 mAb (Zhao et al., 2018).

The exhaustion of lymphocytes, a hypofunctional state characterized by progressive loss of lymphocytes functions and self-renewal capacity, is considered as a pathway of resistance for anti-PD-1 immunotherapy (Chow et al., 2022). In this study, we found that PD-1 mAb can promote the exhaustion of activated T/NK cells *in vitro* co-culture system (Figs. 2C, 4E and 4F). Notably, some researchers also found that CD69 deficient intratumoral T cells showed a decreased proportion of exhaustion in 4T1 tumor-bearing mice (Mita et al., 2018). All the evidences imply that CD69 plays a crucial role in inducing exhaustion of activated lymphocytes. A possible explaination is that PD-1 mAb may trigger the interaction between CD69 molecule expressed on TILs and unknown ligands on tumor cells. Subsequently, continuous tumor antigen stimulations lead to the exhaustion of CD69 positive TILs.

A previous report found that neuroendocrine neoplasm patients with lower pre-treatment activation state (CD69 expression) of naïve T cells and NK cells were associated with longer progression-free survival after anti-PD-1 immunotherapy (MacFarlane et al., 2021). Similarly, our results suggested that the high activation state (CD69^high^) of peripheral T cells and NK cells could be used as a predictive biomarker for acquired resistance of anti-PD-1 immunotherapy. Although our observations are from a very specific *in vitro* model system tempers the strength of related conclusion somewhat, all the consistent findings imply the probability of measuring CD69 expression level as a biomarker for informing and stratifying patients about likely successful response to anti-PD-1 immunotherapy. Our next work would be to interrogate tumor gene expression datasets of NSCLC patients undergoing anti-PD-1 treatment to observe whether CD69 expression level correlates with clinical response rate, or resistance to therapy prior to or at different stages of treatment, and further explain the relevant mechanism.

## Conclusions

Taken together, our results indicated that PD-1 mAb increase the death and exhaustion of CD69^high^ T cells and NK cells in a dose-dependent way, and provided a hypothesis that the high activation state (CD69^high^) of T cells and NK cells may limit the durative efficacy of PD-1 mAbs and lead to occurrence of acquired resistance. These data may provide ideas to guide individualized medication of PD-1 mAb in NSCLC patients.

## Supplemental Information

10.7717/peerj.15374/supp-1 Supplemental Information 1 Supplementary Figures.

## Additional Information and Declarations

### Data Availability

The following information was supplied regarding data availability: The raw flow cytometry data is available at Figshare: Wang, Ying (2023): #82237 The Raw Data.rar. figshare. Dataset. https://doi.org/10.6084/m9.figshare.22083266.v1. Drag the corresponding FlowJoWorkSpace file to the FlowJo software to see the corresponding gates and datas. The FlowJo software is available at: https://www.flowjo.com/solutions/flowjo/downloads.
