# Supplementary material for: Increased death and exhaustion of CD69high T cells and NK cells are associated with PD-1 antibody application in the in vitro co-culture system
Source: PeerJ. 2023 May 8;11:e15374. doi: 10.7717/peerj.15374 (PMC10174060; doi:10.7717/peerj.15374)
Supplement: Supplemental Information 1 [file peerj-11-15374-s001.zip › Supplemental Figure/Supplementary Figure 1.docx]

**Supplementary Figure 1**


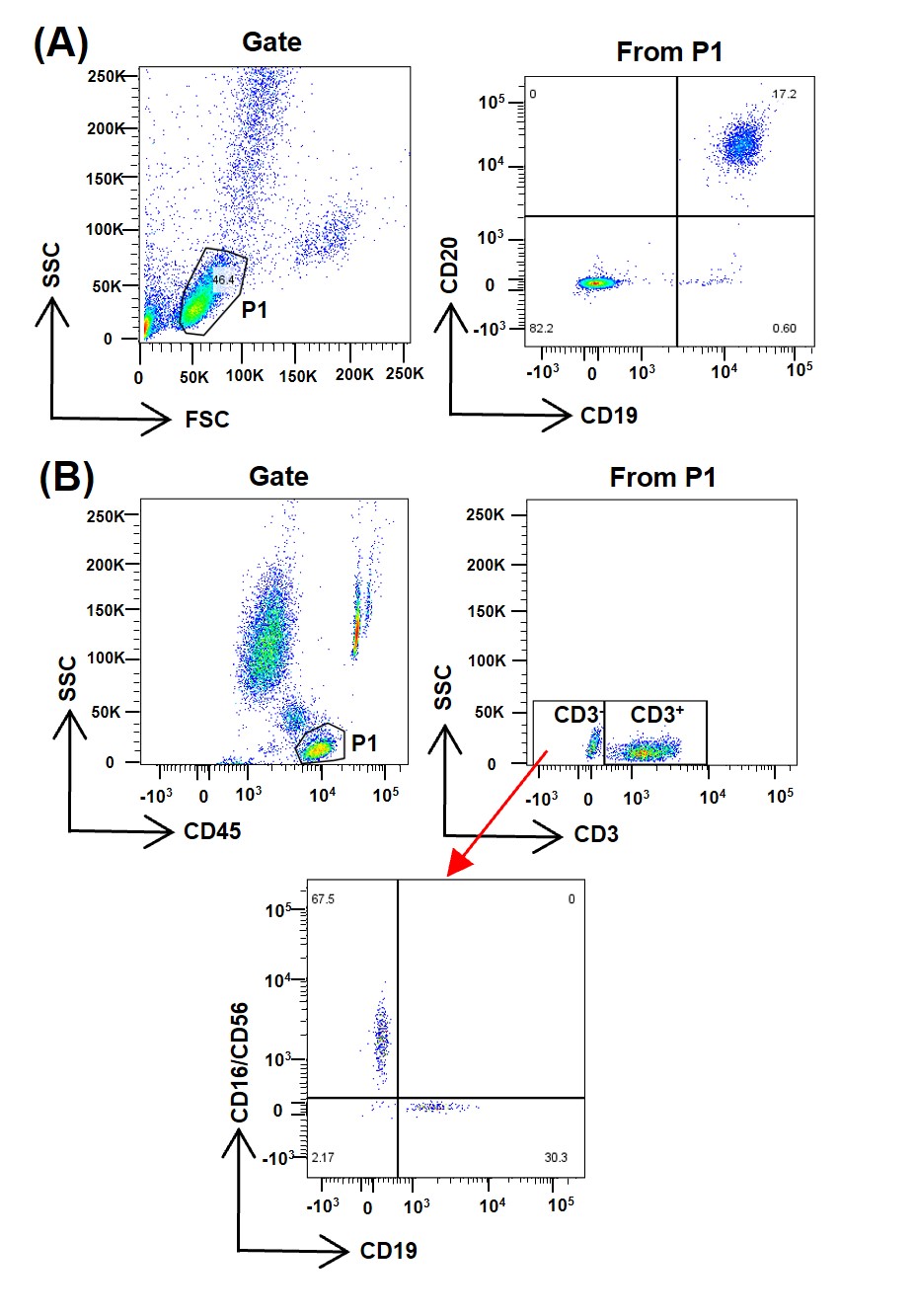


**Supplementary Figure 1. The equivalence analysis of alternative markers or criteria.** (A) The similarity of CD19^+^ B cells and CD20^+^ B cells were analyzed in peripheral blood leukocytes by flow cytometry. P1 gates peripheral lymphocytes. (B) The similarity of CD45^+^CD3^-^CD19^-^ cells and CD45^+^CD3^-^CD56^+^/CD16^+^ NK cells were analyzed in peripheral blood leukocytes by flow cytometry.
