# Supplementary material for: Increased death and exhaustion of CD69high T cells and NK cells are associated with PD-1 antibody application in the in vitro co-culture system
Source: PeerJ. 2023 May 8;11:e15374. doi: 10.7717/peerj.15374 (PMC10174060; doi:10.7717/peerj.15374)
Supplement: Supplemental Information 1 [file peerj-11-15374-s001.zip › Supplemental Figure/Supplementary Figure 2.docx]

**Supplementary Figure 2**


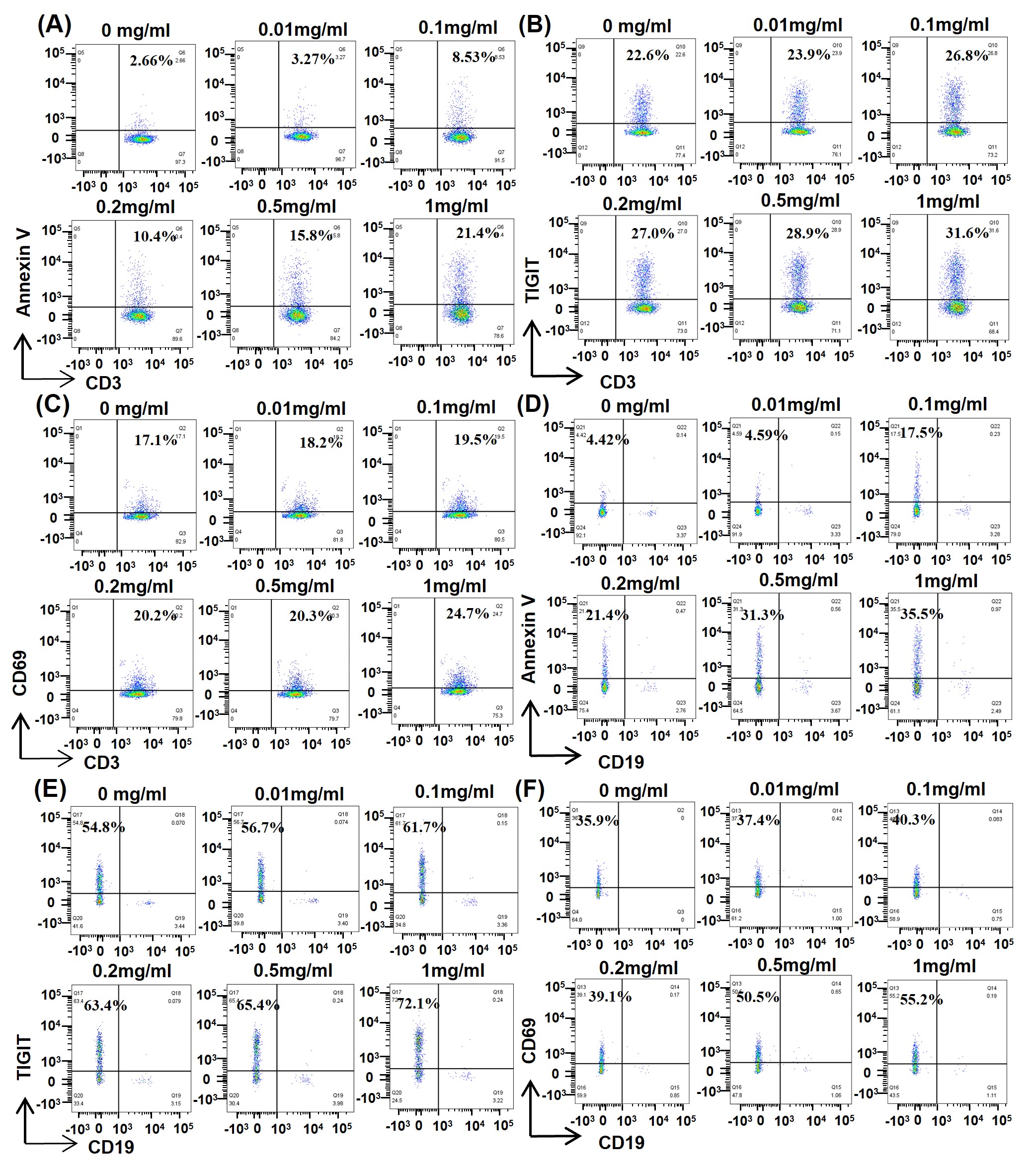


**Supplementary Figure 2.** **For partial NSCLC patients, the death rate and exhaustion of lymphocyte in tumor microenvironment increased with increasing concentration of PD-1 mAb.** PBMCs were isolated from peripheral blood of patients with NSCLC and inoculated into HCC827 cell culture wells with gradually increasing concentration of PD-1 mAb for 24h. Flow-cytometry dot plots show the percentages of Annexin V^+^, TIGIT^+^ and CD69^+^ on T cells and NK cells. (A-C) The expression of (A) Annexin V, (B) TIGIT and (C) CD69 were analyzed on CD45^+^CD3^+^ T cells. (D-F) The expression of (D) Annexin V, (E) TIGIT and (F) CD69 were analyzed on CD45^+^CD3^-^CD19^-^ NK cells.
