# Supplementary material for: Increased death and exhaustion of CD69high T cells and NK cells are associated with PD-1 antibody application in the in vitro co-culture system
Source: PeerJ. 2023 May 8;11:e15374. doi: 10.7717/peerj.15374 (PMC10174060; doi:10.7717/peerj.15374)
Supplement: Supplemental Information 1 [file peerj-11-15374-s001.zip › Supplemental Figure/Supplementary Figure 3.docx]

**Supplementary Figure 3**


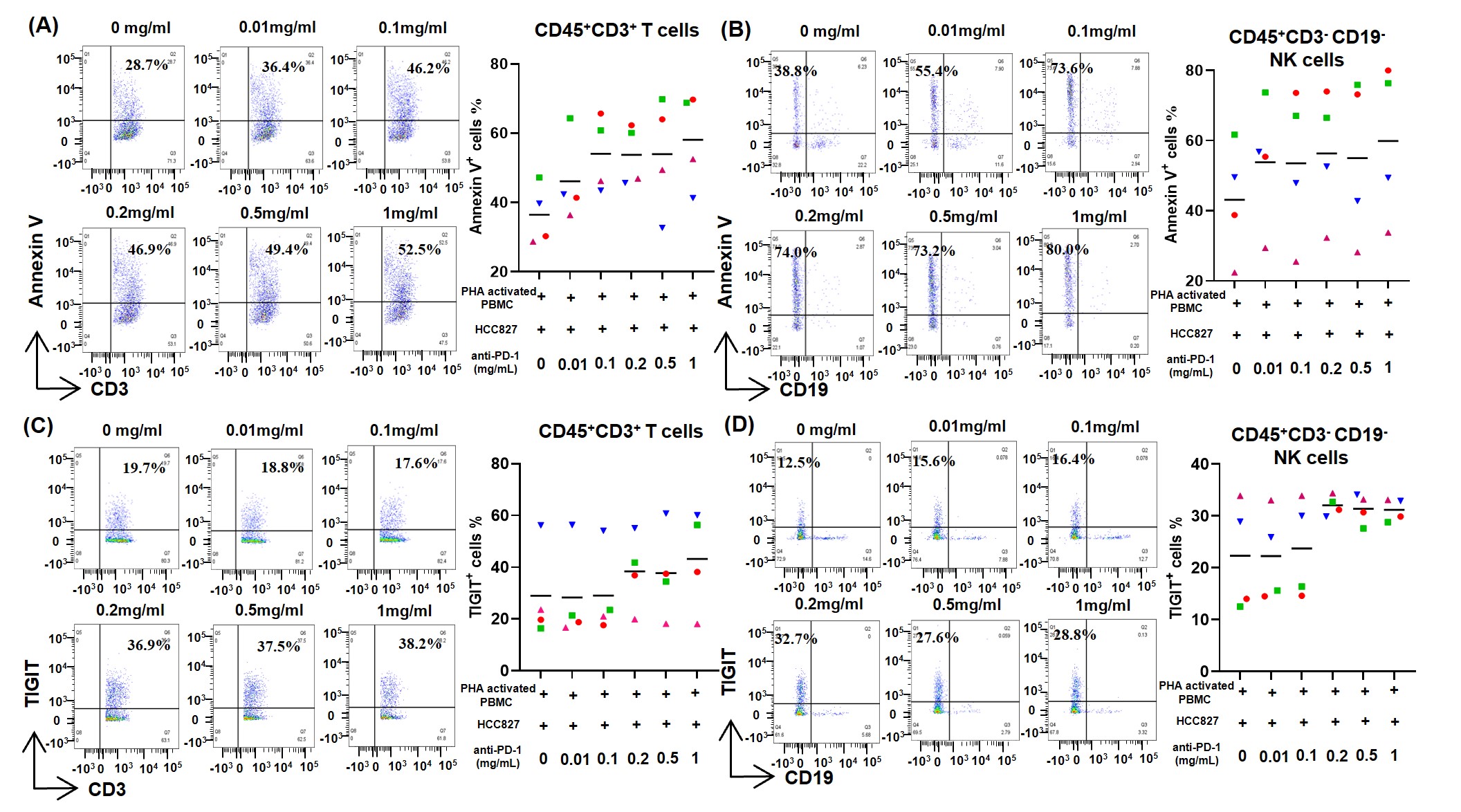


**Supplementary Figure 3. Effects of PD-1 mAb on the death and exhaustion of the PHA activated T cells and NK cells derived from healthy volunteers.** PBMCs were isolated from peripheral blood of four healthy volunteers and treated with 2ug/ml PHA for 24 hours. After washing, they were inoculated into HCC827 cell culture wells in a ratio of 2:1. The gradient increasing concentration of PD-1 mAb were respectively added into co-culture system containing PHA activated PBMCs and HCC827 cells. Flow cytometry analyzed the proportions of (A) CD45^+^CD3^+^Annexin V^+^ T cells and (B) CD45^+^CD3^-^CD19^-^Annexin V^+^ NK cells in different groups. Proportions of (C) CD45^+^CD3^+^TIGIT^+^ T cells and (D) CD45^+^CD3^-^CD19^-^TIGIT^+^ NK cells in different groups. The same shape comes from the same source.
