# Supplementary material for: Increased death and exhaustion of CD69high T cells and NK cells are associated with PD-1 antibody application in the in vitro co-culture system
Source: PeerJ. 2023 May 8;11:e15374. doi: 10.7717/peerj.15374 (PMC10174060; doi:10.7717/peerj.15374)
Supplement: Supplemental Information 1 [file peerj-11-15374-s001.zip › Supplemental Figure/Supplementary Figure 4.docx]

**Supplementary Figure 4**


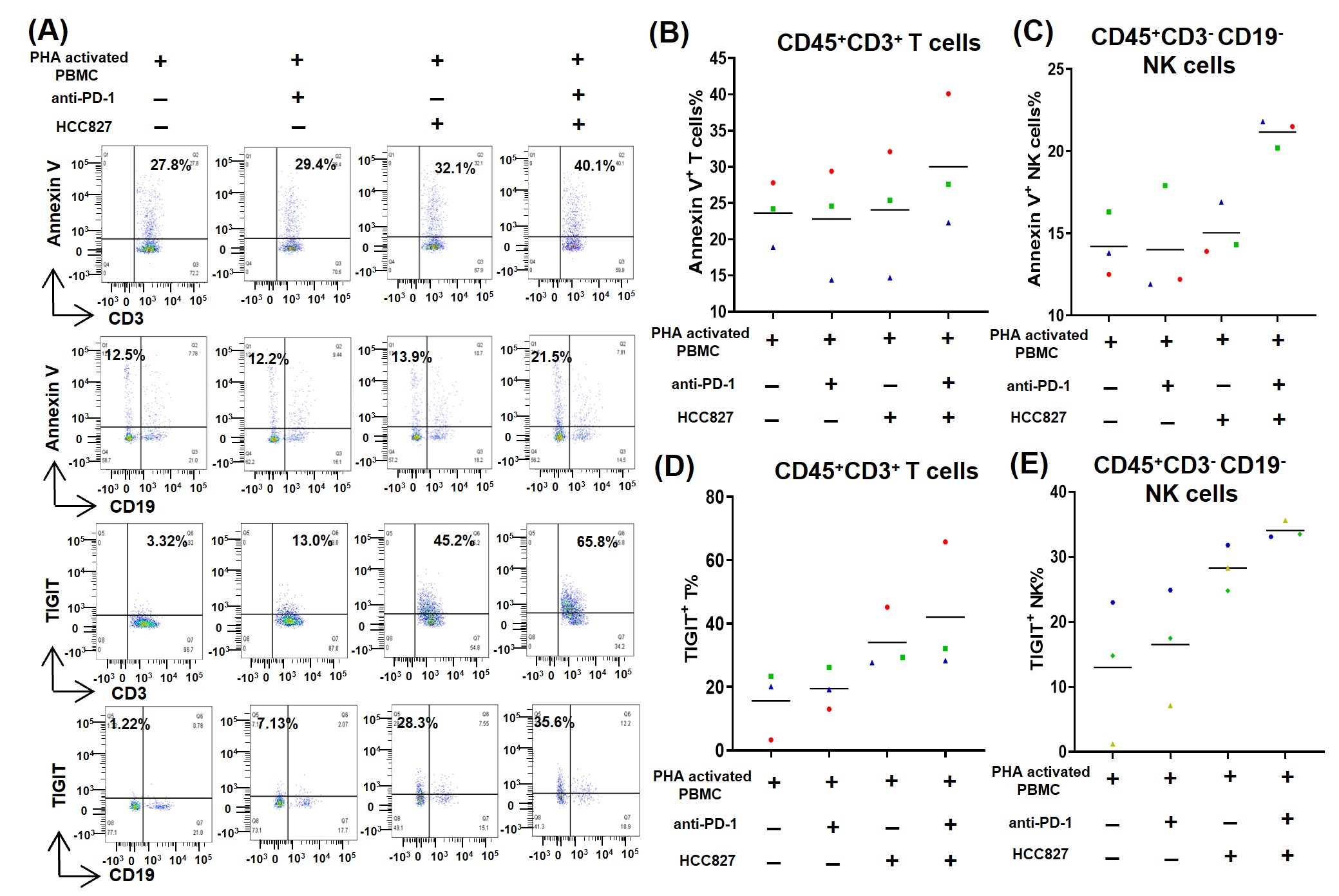


**Supplementary Figure 4. Effects of PD-1 mAb on the death and exhaustion of the activated T cells and NK cells when PBMCs cultured alone or co-cultured with tumor cells.** (A) Flow-cytometry dot plots show the expression of Annexin V and TIGIT on CD45^+^CD3^+^ T cells and CD45^+^CD3^-^CD19^-^Annexin V^+^ NK cells in different groups. (B-E) Analyzing the percentages of Annexin V^+^ and TIGIT^+^ on the PHA activated T and NK cells in different groups. Experiments depicted are representative of 3 performed. The same shape comes from the same source.
